# AllerGAtlas 1.0: a human allergy-related genes database

**DOI:** 10.1093/database/bay010

**Published:** 2018-02-22

**Authors:** Jinying Liu, Yuan Liu, Dan Wang, Mengqi He, Lihong Diao, Zhongyang Liu, Yang Li, Li Tang, Fuchu He, Dong Li, Shuzhen Guo

**Affiliations:** 1 School of Chinese Medicine, Beijing University of Chinese Medicine, Beijing, China; 2 State Key Laboratory of Proteomics, Beijing Proteome Research Center, National Center for Protein Sciences-Beijing (PHOENIX Center), Beijing Institute of Lifeomics, Beijing, China

## Abstract

Allergy is a detrimental hypersensitive response to innocuous environmental antigen, which is caused by the effect of interaction between environmental factors and multiple genetic pre-disposition. In the past decades, hundreds of allergy-related genes have been identified to illustrate the epidemiology and pathogenesis of allergic diseases, which are associated with better endophenotype, novel biomarkers, early-life risk factors and individual differences in treatment responses. However, the information of all these allergy-related genes is dispersed in thousands of publications. Here, we present a manually curated human allergy-related gene database of AllerGAtlas, which contained 1195 well-annotated human allergy-related genes, determined by text-mining and manual curation. AllerGAtlas will be a valuable bioinformatics resource to search human allergy-related genes and explore their functions in allergy for experimental research.

**Database URL**: http://biokb.ncpsb.org/AlleRGatlas/

## Introduction

Allergy is a detrimental immunological hypersensitive response to innocuous environmental antigen (1). By definition, allergy includes in form of various heterogeneous conditions such as anaphylaxis, allergic asthma, atopic dermatitis (AD) and the colorful spectrum of food- or drug–induced hypersensitivity reactions (2, 3). Allergy is characterized as the new epidemic of the 21st century due to the continuous rise in the prevalence and severity (4, 5). According to *The World Allergy OrganizationWhite Book on Allergy*, up to 40% of the population has suffered from one or more type of allergy with significant associated medical and financial burden (6). From 1992 to 2012, there was a 615% increase in the rate of hospital anaphylaxis admission in the UK, with an estimated annual cost of €900 million (7, 8).

Allergies are clinically and genetically heterogeneous diseases with a variable clinical course and with important divergences in the response to therapy, which can lead to significant challenges for the correct diagnosis and proper treatment. Although allergies can be classified into distinct disorder categories, they show clinical overlap and share common genetic risk factors. For example, allergic asthma or AD, is unlikely to be a single disease but rather a series of overlapping individual clinical features or phenotypes with unique genetic and environmental contributors (9, 10). There is a growing consensus that allergy is caused by the effect of interaction between environmental factors and multiple genetic pre-disposition (11, 12). Therefore, it is important to understand how associated genetic and environmental factors increase the complexity of allergic disease.

Hundreds of allergy-related genes have been discovered, which are involved in better endophenotype, identification of at-risk individuals in early life, novel biomarkers and individual treatment responses. For example, FLG (filament aggregating protein) has already been proposed as a robust screening biomarker for early-onset severe AD, which also has highlighted the importance of epidermal barrier dysfunction in the development of allergic diseases (13, 14). The gene polymorphisms of cluster of differentiation 14, toll-like receptors, Glutathione S-transferase Mu 1 and Glutathione S-Transferase Pi 1 (15) have shed light on the importance of gene–environment interaction for allergic diseases (16–18). Serum levels of Interleukin 16, Interleukin 31, eosinophil cationic protein and High mobility group box 1 can reflect and stratify diseases severity and have been used as reliable markers (19–21). Studies of associations between variants in Adrenoceptor Beta 2, Arachidonate 5-lipoxygenase, IL-4 Receptor Subunit Alpha

 and response to related pharmaceuticals are crucial for precision medicine (22–24). Recently, large numbers of genome wide association studies aiming to explore genetic susceptibility have accelerated the search for novel and interesting genes for human allergy-related disease. For example, Bønnelykke *et al*. (25) increased the number of susceptibility loci from 3 to 10 with replication in 6114 case individuals and 9920 controls by the first large-scale genome-wide association study, including single nucleotide polymorphisms (SNPs) in or near TLR6, C11orf30, STAT6, SLC25A46, HLA-DQB1, IL1RL1, LPP, MYC, IL2 and HLA-B. In a large-scale GWAS on 11 025 AD cases and 40 398 controls, Paternoster *et al*. (26) identified and replicated two novel SNPs related to epidermal proliferation and differentiation (rs479844 upstream of OVOL1 and rs2164983 near ACTL9). Ramasamy *et al*. (27) identified three common genetic variants (rs7775228, rs2155219, rs17513503) associated with prevalent allergic rhinitis and grass sensitization, by using approximately 2.2 million genotyped or imputed SNPs in four large European adult cohorts for allergic rhinitis (3933 self-reported cases vs 8965 control subjects) and grass sensitization (2315 cases vs 10 032 control subjects). Studies also identified new genetic susceptibility factors and suggested previously unidentified biological pathways associated with allergic diseases in ethnically different populations, such as rs7701890and rs6010620 in the Chinese sample, rs6010620 in the German sample, as well as rs2243250 and rs2227284 in Pakistani sample (28, 29).

However, the rich scientific information about previously identified allergy-related genes is dispersed in thousands of publications. There is still no database focusing on allergy-related genes so far, which presents the obstacle to understand the genetic architecture of allergic diseases. To address this need, we build the AllerGAtlas database 1.0 (http://biokb.ncpsb.org/AllerGAtlas/) that collects 1195 identified allergy-related genes by the literature-mining and manual curation. AllerGAtlas will lead to new insights into the pathogenesis and epidemiology of individual cases, novel diagnostic and prognostic biomarker, individual treatment responses and precision medicine.

## Literature mining and manual curation

To obtain a complete list of allergy-related genes, we performed a comprehensive search for allergy-related literature abstracts in PubMed. Gene-nomenclature recognition and extraction from these abstracts for human allergy-related gene candidates were performed by self-developed ontology-based bio-entity recognizer, which has the precision, recall, F-measure of 0.810, 0.883, 0.845 against the CRAFT corpus for gene/protein recognition based on Protein Ontology (PR) and is on par with current state-of-the-art biomedical annotation systems like BeCAS (30).

A list of human allergy-related genes together with their related diseases and evidence from PubMed abstract was compiled in the following three steps. First, 242 066 sentences in 112 979 PubMed abstracts containing the keywords of ‘allergy,’ ‘allergic,’ ‘anaphylaxis,’ ‘allergic reaction,’ ‘allergic response,’ ‘hypersensitivity,’ ‘atopic,’ ‘atopic’ or their lexical variants were collected. Second, a list of 3150 human genes co-occured with the allergy-related keywords at single-sentence level were recognized and extracted from 42 975 sentences in 27 033 PubMed abstracts by our bio-entity recognizer based on PR. Third, all these 3150 candidates were manually curated by our experts and 1195 genes were finally identified as human allergy-related genes.

The human allergy related disease terms were identified from PubMed abstracts by bio-entity recognizer based on Human Disease Ontology (DO) (31). Associations between allergy-related genes/proteins and human disease terms were obtained based on sentence-level co-occurrence. Furthermore, the biomarker of certain genes/proteins are recognized and marked with the keywords of either ‘biomarker’ or ‘marker’ or their lexical variants like ‘biomarkers,’ ‘markers,’ ‘mark’ and ‘biochemical markers.’

### Genes annotation

With the purpose to facilitate deeper interpretations of the relations with allergy, we provided detailed annotations for each gene. NCBI Entrez Gene ID or gene symbol were used as the central ID for cross-linking and annotation. The basic gene annotation files were downloaded from NCBI FTP site. The gene_info and gene2refseq files were parsed to extract the basic gene information such as gene symbol, synonyms, full name, genetic location, gene type, chromosome, chromosomal location and reference sequence information. The gene ontology (GO) annotations for each gene were obtained from the GOA database (32) and the gene-pathway mapping relations were downloaded from Reactome database (33). The public database dbSNP (34) was utilized to map SNPs to genes by the literature’s PMIDs (PubMed Unique Identifier). Public databases including Ensembl (35), Entrez gene (36), UniProt (37), neXtProt (38), Anti-bodypedia (39) also were used for mapping and annotating.

## Database implement and service status

All extracted allergy-related genes/proteins, human disease terms as well as their biomarker roles were loaded into a local MySQL database. PHP was used to build the website of AllerGAtlas on a Windows server. The web service is available at http://biokb.ncpsb.org/AllerGAtlas/. All the data of AllerGAtlas is available to all users without login or registration.

## Database search and navigation

**Figure 1.**
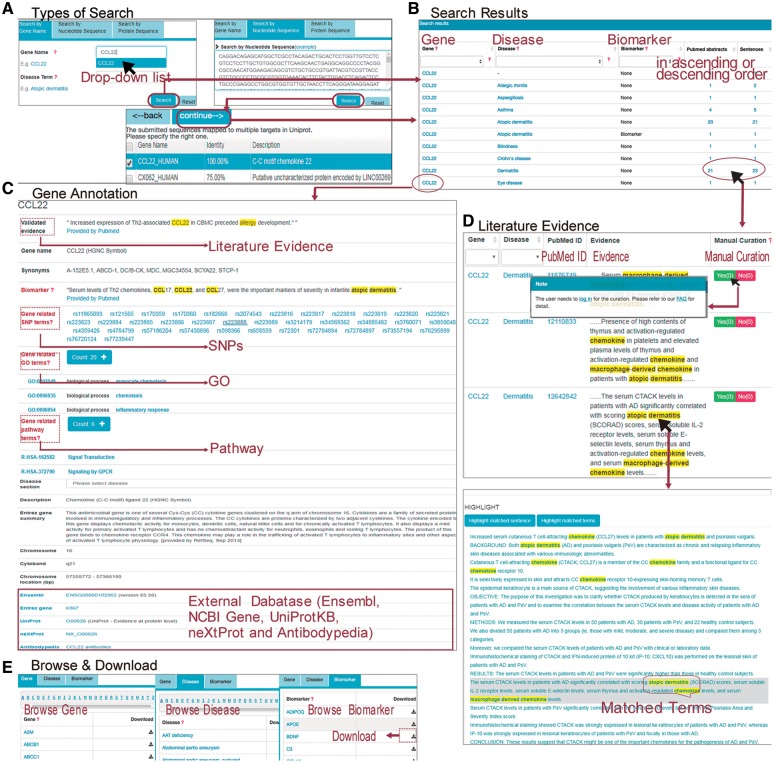
(**A**) The page of ‘Home’ supports three types of queries: search by gene name, search by nucleotide or protein sequence. Users can submit the gene name of ‘CCL22’ in the search box. Users can also search the gene by sequence, and the sequence identity score from BLAST will be listed. Users can specify the matched gene symbol and click ‘continue’ for result page. (**B**) On the result page, a table containing the queried gene associated human disease terms and supporting evidence is displayed. (**C**) After clicking the gene name of ‘CCL22’ in the page of ‘result,’ users can see detailed annotation about this gene and cross references to external databases. (**D**) After clicking the number of the evidence abstracts or sentences in the page of ‘result,’ users can see a table containing the gene, associated disease term, the PubMed ID, the evidence sentence and the manual validation information. Users can also click on the link of evidence in this page to see the original abstract highlighted with the key words. (**E**) The page of ‘Browse & Download’ presents three different approaches for browsing. All the information can be downloaded.

AllerGAtlas provides a user-friendly web interface that facilitates searching and browsing database (http://biokb.ncpsb.org/aagatlas/), which comprises five sections including ‘Home,’ ‘Browse & Download,’ ‘Feedback,’ ‘FAQ’ and ‘Contact’ (Figure 1). In the page of ‘Home,’ users can search AllerGAtlas 1.0 database by three types of queries: protein name, nucleotide sequence and protein sequence. For the gene name query, the user can input a gene name in the search box of ‘Gene Symbol,’ and a drop down list with auto-completed gene symbols will be present in the AllerGAtlas. After selecting one of them and clicking the ‘Search’ button, the search engine will run and return the queried results containing the queried gene associated human disease terms and supporting literature evidence. If you search the gene by nucleotide sequence or protein sequence, the sequence identity score from BLAST will be listed. Users can specify the matched gene symbol and click ‘continue’ for result page (Figure 1A). On the result page, a table containing the queried gene, associated human disease terms and number of the supporting abstracts or sentences is displayed (Figure 1B). The hyperlink of the queried gene can lead to the gene info page with plenty of information, including a list of SNP terms from dbSNP, GO terms from GOA, pathway terms from Reactome, the protein description from UniProtKB, the gene expression info from the Expression Atlas, the protein expression info from Human Protein Atlas and the related disease info from Human Disease Ontology (Figure 1C). The hyperlink of number of the evidence abstracts or sentences can lead to a table containing the gene, the disease, the PubMed ID, the evidence sentence and the manual validation information. In addition, the hyperlink of an individual interested evidence sentence leads to the whole abstract with the supporting sentence and various types of extracted entity terms highlighted, i.e. gene name and disease terms (Figure 1D). Our website supports three different approaches for browsing by the page of ‘Browse & Download.’ All the information can be downloaded (Figure 1E).

### Application case of the database

**Figure 2.**
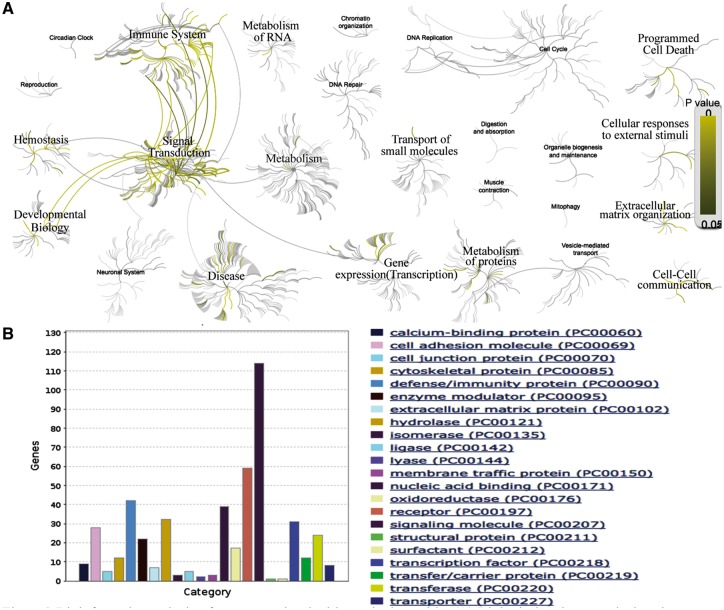
Bioinformatics analysis of genes associated with AD. (**A**) Biological pathway analysis using Reactome (http://www.reactome.org/). (**B**) Protein class analysis using PANTHER (http://pantherdb.org/).

AD is the most common skin disease, affecting up to 30% of children and 3% of adults worldwide (40). We searched our database with the disease name of ‘AD’ and found a list of 538 expert curated genes with detailed annotations. To obtain more detailed functional annotation of each gene, we can click the hyperlink of the related genes which can lead to the gene info page with plenty of information, such as gene related SNP, gene related pathway and gene related GO terms. Further, we can perform the functional and pathway analyses on the list of AD related genes. Analysis with Reactome (http://www.reactome.org/) reveals that these genes tend to participate in the pathways of immune system, signal transduction, gene expression (transcription), metabolism of proteins, developmental biology, hemostasis, cell–cell communication, extracellular matrix organization, cellular responses to external stimuli and programmed cell death (Figure 2A). The protein class analysis with Panther (41) (http://pantherdb.org/) indicates that these genes tend to be with the function of signaling molecule, receptor, defense/immunity protein, nucleic acid binding, hydrolase, transcription factor, cell adhesion molecule, transferase, etc. (Figure 2B). This result suggests that the interaction between innate/adaptive immune responses and skin epithelial function play a major role in the development of AD. The above speculation can be validated by the literature (42).

## Discussion

**Figure 3.**
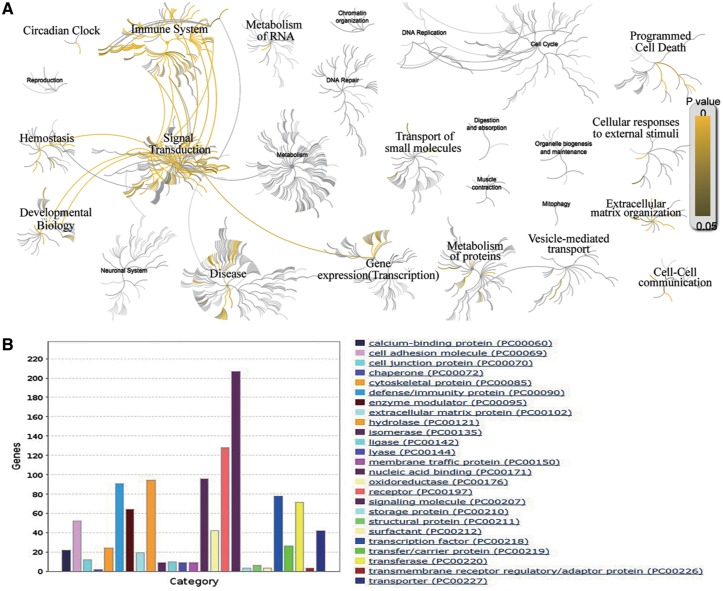
Bioinformatics analysis of the human allergy-related genes. (**A**) Biological pathway analysis using Reactome. (**B**) Protein class analysis using PANTHER.

The comprehensive collection of AllerGAtlas 1.0 database allows us to have an overview of human allergy-related genes and their related diseases. The analysis with Reactome reveals that these genes participate in the immune system, signal transduction, transcription, metabolism of proteins, developmental biology, hemostasis, vesicle-mediated transport, cell–cell communication, extracellular matrix organization, cellular responses to external stimuli, programmed cell death, transport of small molecules, metabolism of RNA and circadian clock (Figure 3A). The GO analysis with Panther reveals that the largest class of allergy-related gene proteins is signaling molecule, followed by receptor, nucleic acid binding, hydrolase, defense/immunity protein, transcription factors, transferase, enzyme modulator, cell adhesion molecule, etc (Figure 3B). All these results show the importance and the value of our collection, as well as demonstrate that AllerGAtlas 1.0 database will greatly facilitate allergist to explore the pathogenesis of allergies.

The web service of AllerGAtlas 1.0 also supports the function of community curation. All logged in users can provide their feedback by simply clicking the ‘Yes’ or ‘No’ button to confirm or reject the evidence phrases. Our database will be updated periodically according to these feedbacks.

In summary, AllerGAtlas 1.0 is the first attempt to provide a comprehensive non-redundant catalog of allergy-related genes along with supporting evidence from published literature. The availability and use of AllerGAtlas 1.0 will be expected to be a unique value-added resource, which can help scientists and clinicians to search the literature on allergy-related genes and their involvement in diseases.

## Funding

This work was supported by the Program of the National Key Research and Development Program (2017YFC1700105), Program of International S&T Cooperation (2014DFB30020), Innovation Project (16CXZ027), Beijing Nova Program (xx2014009), National Natural Science Foundation of China (31601064) and the Beijing Nova Program (Z171100001117117), Program of Precision Medicine (2016YFC0901905). 

*Conflict of interest*. None declared.
